# Early Onset Prion Disease from Octarepeat Expansion Correlates with Copper Binding Properties

**DOI:** 10.1371/journal.ppat.1000390

**Published:** 2009-04-17

**Authors:** Daniel J. Stevens, Eric D. Walter, Abel Rodríguez, David Draper, Paul Davies, David R. Brown, Glenn L. Millhauser

**Affiliations:** 1 Department of Chemistry and Biochemistry, University of California Santa Cruz, Santa Cruz, California, United States of America; 2 Department of Applied Mathematics and Statistics, University of California Santa Cruz, Santa Cruz, California, United States of America; 3 Department of Biology and Biochemistry, University of Bath, Bath, United Kingdom; Istituto Superiore di Sanità, Italy

## Abstract

Insertional mutations leading to expansion of the octarepeat domain of the prion protein (PrP) are directly linked to prion disease. While normal PrP has four PHGGGWGQ octapeptide segments in its flexible N-terminal domain, expanded forms may have up to nine additional octapeptide inserts. The type of prion disease segregates with the degree of expansion. With up to four extra octarepeats, the average onset age is above 60 years, whereas five to nine extra octarepeats results in an average onset age between 30 and 40 years, a difference of almost three decades. In wild-type PrP, the octarepeat domain takes up copper (Cu^2+^) and is considered essential for in vivo function. Work from our lab demonstrates that the copper coordination mode depends on the precise ratio of Cu^2+^ to protein. At low Cu^2+^ levels, coordination involves histidine side chains from adjacent octarepeats, whereas at high levels each repeat takes up a single copper ion through interactions with the histidine side chain and neighboring backbone amides. Here we use both octarepeat constructs and recombinant PrP to examine how copper coordination modes are influenced by octarepeat expansion. We find that there is little change in affinity or coordination mode populations for octarepeat domains with up to seven segments (three inserts). However, domains with eight or nine total repeats (four or five inserts) become energetically arrested in the multi-histidine coordination mode, as dictated by higher copper uptake capacity and also by increased binding affinity. We next pooled all published cases of human prion disease resulting from octarepeat expansion and find remarkable agreement between the sudden length-dependent change in copper coordination and onset age. Together, these findings suggest that either loss of PrP copper-dependent function or loss of copper-mediated protection against PrP polymerization makes a significant contribution to early onset prion disease.

## Author Summary

Prion diseases are neurodegenerative disorders involving the prion protein, a normal component of the central nervous system. An unusual class of inherited mutations giving rise to prion disease involves elongation of the so-called octarepeat domain, near the protein's N-terminus. Research from our lab and others shows that this domain binds the micronutrient copper, an essential element for proper neurological function. We investigated how octarepeat elongation influences copper binding by examining both the molecular features and the binding equilibrium. We find that elongation beyond a specific threshold, which confers profound early onset disease, gives rise to concomitant changes in copper uptake. The remarkable agreement between onset age and altered copper binding points to loss of copper protein function as significant in prion neurodegeneration.

## Introduction

**Figure 1:**
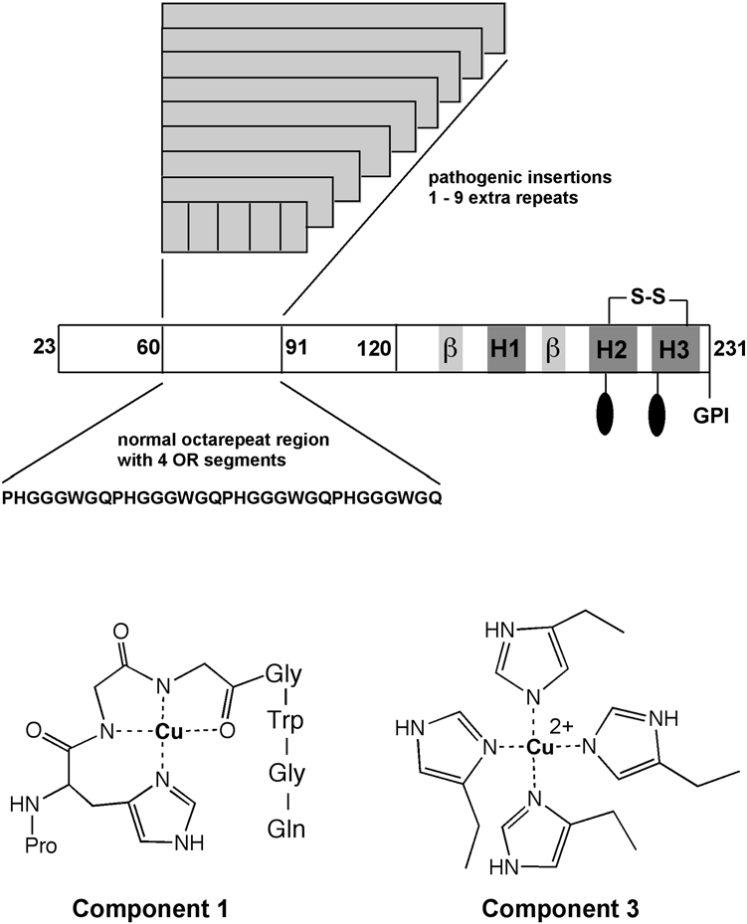
Schematic of PrP(23–231) showing placement of the octarepeat region within the flexible N-terminal domain (23–120). Wild-type PrP has four repeats, as indicated by the octarepeat sequence. Low copper occupancy favors component 3 coordination; high copper occupancy favors component 1. Mutations involving insertion of extra (1–9) octarepeats are pathogenic. Other indicated PrP features are the C-terminal β-strands, helical segments (H1–H3), disulfide bond, GPI anchor, and N-linked glycans (ovals).

Prion diseases are infectious neurodegenerative disorders that arise from accumulation of PrP^Sc^ (scrapie conformer), a misfolded form of the normal cellular prion protein (PrP^C^) that is found ubiquitously throughout the central nervous system [1]–[3]. PrP^C^ is a GPI anchored glycoprotein possessing a largely α-helical C-terminal domain and a flexible N-terminal domain (Figure 1). Within the N-terminal domain are four tandem copies of the octapeptide repeat (octarepeat) sequence PHGGGWGQ. Approximately 15% of human prion diseases are inherited [4]. The known disease-causing mutations are either point mutations, located primarily in the C-terminal domain, or insertions of one to nine extra octarepeats resulting in expansion of the N-terminal domain [5]. Interestingly, with octarepeat expansion disease, progression is determined by the number of repeat inserts. With one to four extra octarepeats, the average onset age is 64 years, whereas five to nine extra octarepeats results in an average onset age of 38 years, a difference of almost three decades [5].

Octarepeat (OR) expansions alter the properties of PrP and its interactions with cellular components. When expressed in various cell lines, PrP with additional repeats displays detergent insolubility, resistance to proteinase K digestion similar to PrP^Sc^ [6], altered cell surface expression [7], and hindered export to the cell surface [8]. Moreover, compared to wild-type, expanded PrP exhibits a stronger association with the cell membrane and a larger proportion of partially glycosylated forms [9]. Transgenic mice expressing insert mutants of PrP develop prion disease and show accumulation of detergent-insoluble, protease-resistant PrP in the brain [10],[11]. Although injection of brain homogenate from these transgenic mice is not infectious, brain suspensions from humans with insert mutations can transmit disease to monkeys and chimpanzees [12]–[14]. Moreover, *in vitro* assays show that recombinant protein containing insert mutations forms amyloid fibrils faster than wild-type [15]. Truncated forms of the protein with extra octarepeats show irreversible self association and, unlike wild-type, can bind PrP^Sc^ [16]. Full length mouse PrP with expanded OR domains shows an altered folding landscape that reduces the propensity for amyloid formation [17].

After treatment with proteinase K, aggregated PrP^Sc^ typically retains an intact protease-resistant core region, which includes the protein's C-terminal domain, and remains capable of propagating disease [2],[18]. The OR region is located outside of this core portion of the protein and is cleaved away by proteinase K. Octarepeat inserts are therefore the only known disease-causing mutations occurring outside the minimal infectious PrP^Sc^ substructure. This suggests that either disease propagation and mechanisms underlying prion-mediated neurodegeneration are separable or, alternatively, that disease resulting from octarepeat expansions is distinct from other inherited prion diseases.

A notable feature of the octarepeat domain is that it takes up copper ions (Cu^2+^) with an affinity that approximately matches extracellular copper concentrations in the brain [19],[20]. Although the specific function of PrP^C^ is not yet known, the demonstrated interaction with copper suggests a number of possibilities, including protection against Cu^2+^ mediated oxidative stress, copper transport and copper dependent cellular signaling [21]–[23]. In vitro cell culture studies show that Cu^2+^ stimulates PrP endocytosis [24], but this process is quenched in cells expressing insert mutations of nine extra repeats [25]. The way in which PrP coordinates Cu^2+^ depends on the ratio of copper to protein (Figure 1) [26]. At low copper occupancy, the OR domain wraps around a single Cu^2+^ coordinating through multiple His side chains. At high occupancy, each HGGGW segment within an octapeptide coordinates a single Cu^2+^ through the His side chain and deprotonated amides of the following two Gly residues [27]. These coordination modes are referred to as component 3 and component 1, respectively [26].

Previous studies examined the biophysical properties of expanded octarepeat domains with emphasis on either the rate of amyloid production or its uncomplexed backbone conformation [16],[17],[28]. To our knowledge, however, none of these studies has identified a quantitative link between octarepeat length and age of disease onset. Here we use electron paramagnetic resonance (EPR) and affinity studies to examine PrP N-terminal constructs and full-length protein to examine how copper coordinates in the octarepeat domain as a function of domain length. We identify a sharp, length-dependent threshold with regard to coordination mode and affinity. Next, we survey all reported cases of human prion disease resulting from octarepeat expansion and examine age of onset as a function of domain length. We find a remarkable agreement between alteration in copper coordination properties and octarepeat inserts associated with early onset disease.

## Results

### EPR of Wild-Type and Octarepeat Expanded PrP

**Figure 2:**
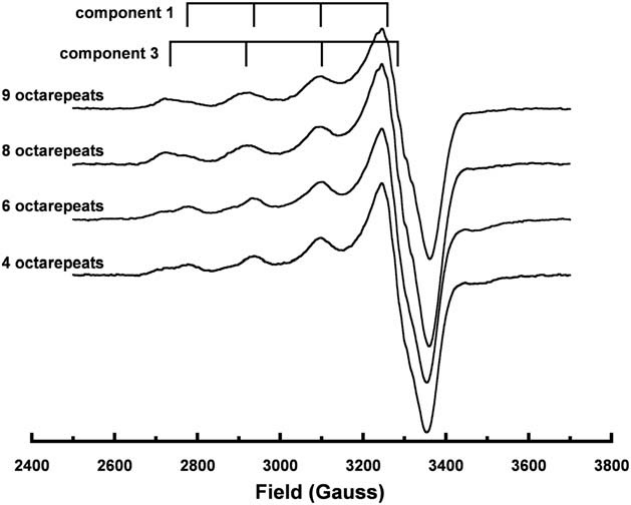
EPR spectra of octarepeat constructs in equilibrium with 3.0 equivalents of Cu^2+^. With four and six octarepeats, the spectra are nearly equivalent and show a mixture of component 1 and component 3 coordination. For longer octarepeat domains, there is a progressive shift to favor component 3 coordination.

Polypeptide constructs corresponding to wild-type and octarepeat insert mutations of up to nine total ORs (Table 1) were synthesized and examined by EPR. Following our previous studies, each construct begins with the pentapeptide segment corresponding to residues 23–27 from PrP, to improve solubility in aqueous solution, and is N-terminally acetylated to prevent spurious Cu^2+^ coordination. Figure 2 shows EPR obtained from each OR domain construct in equilibrium with 3.0 equivalents of Cu^2+^. Hyperfine splittings in the parallel region of the spectra are diagnostic for the different binding modes, as indicated. The 4 OR construct, corresponding to wild-type, exhibits both component 1 and component 3 coordination. However, with increasing length of the OR domain, the equilibrium distribution shifts to favor predominantly component 3 coordination.

**Table 1:** Peptide Sequences.

Ac-KKRPK(PHGGGWGQ)_4_	corresponding to PrP(23–27,60–91)
Ac-KKRPK(PHGGGWGQ)_6_	
Ac-KKRPK(PHGGGWGQ)_7_	
Ac-KKRPK(PHGGGWGQ)_8_	
Ac-KKRPK(PHGGGWGQ)_9_	

**Figure 3:**
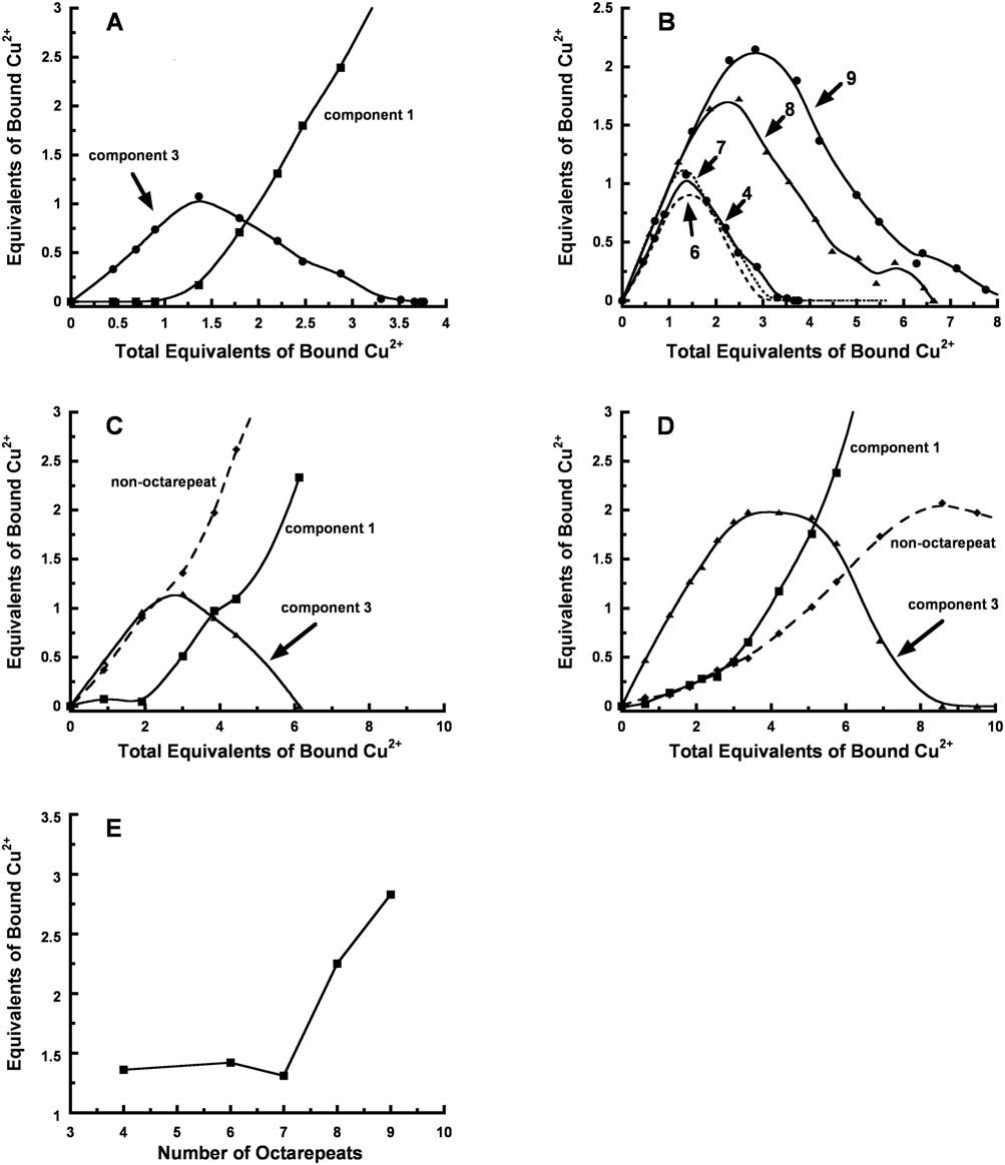
Copper coordination properties as a function of octarepeat domain length. (A) Relative populations of components 1 and 3 in the wild-type octarepeat segment (four repeats). The x-axis reflects the total bound Cu^2+^ equivalents, while the y-axis is for each individual binding component. Component 3 is at a maximum at 1.0–1.5 equivalents bound. (B) Component 3 populations in octarepeat sequences of increasing length. Beyond a threshold of seven repeats, component 3 becomes persistent. (C,D) Component populations in wild-type rPrP and rPrP+5OR, respectively. (E) Persistence of component 3 as measured by the location of the respective maxima from (B).

To determine the relative concentrations of the different binding modes for each construct, we used non-negative least squares (NNLS) fitting to a set of well characterized basis spectra, as previously described [19]. Figure 3A shows that when the 4 OR construct is titrated with Cu^2+^, the populations shift systematically depending on the specific Cu^2+^ concentration. Initially, component 3 dominates, but beyond 1.0–1.5 equivalents, component 3 diminishes and is replaced by component 1. There is also a low concentration of component 2 (2-His coordination), but this intermediate species is relatively minor. The experiment was repeated for all the expanded OR constructs, and the results for component 3 are shown in Figure 3B. With 4–7 total ORs (i.e., zero to three inserts), the behavior is much like wild-type, reaching a maximum of component 3 at approximately 1.0–1.5 equivalents Cu^2+^. In contrast, the 8 OR construct (four inserts) exhibits persistent component 3 coordination that reaches a maximum at approximately 2.0–2.5 equivalents. For the 9 OR construct, the maximum is shifted to yet higher Cu^2+^ concentration reaching a maximum at 3.0–3.5 equivalents. In addition to changes in the location of the maximum, there is a shift in the amount of Cu^2+^ bound in the component 3 mode. For 4–7 ORs, the maximum is approximately 1.0 equivalents. However, for 8 and 9 ORs, 1.5–2.0 equivalents bind in the component 3 mode.

Titrations were also performed with full-length wild-type recombinant PrP (rPrP) and rPrP containing five extra OR segments to give nine total (rPrP+5OR). Titrations with full-length protein require accounting of both OR binding and non-OR binding (involving two His residues between the octarepeat domain and the folded C-terminus), as shown in Figures 3C and 3D [29]. With 2.0 equivalents of Cu^2+^, rPrP shows approximately equal populations of component 3 and non-OR coordination, at 1.0 equivalent each. At higher copper concentrations, component 3 coordination decreases, followed by an increase in component 1 coordination. The behavior of rPrP closely parallels that of the 4 OR construct in Figure 3A, except that component 3 coordination reaches its peak between 2.0–3.0 equivalents of Cu^2+^ since this coordination mode competes with non-OR binding. The titration of rPrP+5OR, shown in Figure 3D, exhibits a remarkable persistence of component 3 coordination. At 5.0 equivalents of Cu^2+^, component 3 remains the dominant species. In contrast, component 3 in wild-type at 5.0 equivalents Cu^2+^ accounts for only a small fraction (approximately 10%) of the total copper bound species. The shift in the persistence of component 3 coordination is shown in Figure 3E where the maximum of component 3 coordination (derived from the data in Figure 3B) is plotted against OR length. Taken together, these experiments with both OR polypeptides and full-length rPrP show that expanded OR domains exhibit a dramatic shift at eight or more repeats that greatly favors multiple His component 3 coordination relative to wild-type.

### Copper Dissociation Constants of Wild-Type and Octarepeat Expanded PrP

**Figure 4:**
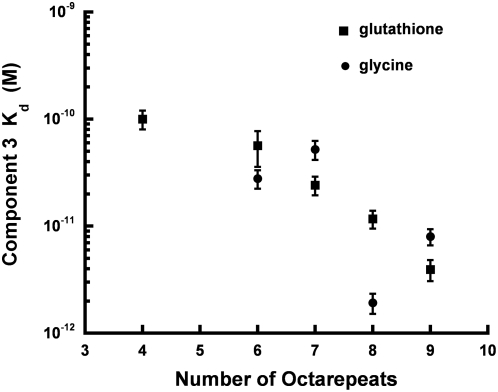
Component 3 dissociation constants for different OR lengths. Values were determined from competition studies with oxidized glutathione or pentaglycine.

Component 3 dissociation constants were measured using a competition assay developed by our lab [19]. Copper binding chelators with known dissociation constants were added to solutions containing OR constructs, along with substoichiometric amounts of Cu^2+^. Decomposition of the resulting EPR spectra reveals the concentration ratios of Cu^2+^ bound to OR construct vs chelator. By working at low copper concentration, we ensure that OR constructs coordinate exclusively as component 3, and this is further verified by lineshape analysis of the EPR spectra. We performed independent measurements with the chelators pentaglycine and oxidized glutathione (two glutathione tripeptides linked through a disulfide bond). *K_d_* values for Cu^2+^ are known for each chelator and are similar to the previously determined dissociation constant for wild-type component 3 [30],[31]. Moreover, these chelators bind Cu^2+^ as a 1∶1 complex, which simplifies the determination of equilibrium constants. The results, as a function of OR length, are reported in Figure 4. For wild-type with four OR segments, the *K_d_* is approximately 10^−10^ M, consistent with our previous results [19]. However, for eight and nine OR constructs, *K_d_* decreases approximately by a factor of 10. There is a systematic difference between oxidized glutathione and pentaglycine, with the latter reporting lower *K_d_* values. Considering the more conservative results from oxidized glutathione, the affinity for Cu^2+^ with component 3 coordination is at least tenfold higher in expanded OR domains with eight or more repeat units.

### Octarepeat Expansion Disease in Humans

Insertions of extra repeats in humans causes prion disease but the course of disease depends on the specific length of the OR domain. Analyses of case studies find consistently that individuals with insertions of five or more ORs often develop symptoms in their 30 s, approximately three decades younger than most instances of sporadic or inherited prion disease [5],[32]. To compare the correlation between OR length and onset age to our biophysical findings, we examined all reported case studies of prion disease arising from OR insertions. Data from Croes et al. [32] and Kong et al. [5], as well as several new case studies were pooled (Table 2). Together, the data of Table 2 represent 31 reports covering approximately 30 families and 108 individuals. Entries are ordered with respect to the number of insertions and, along with each entry, are the range for the age of onset, disease duration and pathology with regard to PrP associated plaques. Many cases that examined tissue pathology identified plaques (although in many instances it was not clear whether reported plaques were amyloid).

**Table 2:** Reported Cases of Extra Repeat Disease.

Reference (First Author)	Number of Inserted Octarepeats (Number of Cases)	Onset Age (Years)	Duration	Cerebellar Plaques
Laplanche [47]	1(1)	73	4 mo	—
Pietrini [48]	1(2)	58/64	5/6 mo	Isolated plaque-like deposits
Van Harten [49]	2(1)	61	7 yr	—
Croes [32]	2(1)	59	>10 yr	—
Grabson-Frodl [50]	3(1)	69	4 mo	Ovoid/round plaque-like deposits
Nishida [51]	3(1)	68	3 yr	—
Laplanche [47]	4(1)	82	4 mo	—
Isozaki [52]	4(1)	62	—	PrP plaques
Campbell [53]	4(1)	56	2 mo	PrP patches
Rossi [54]	4(1)	65	6 mo	Dotted PrP deposits
Yanagihara [55]	4(1)	56	5 mo	—
Goldfarb [14]	5(2)	31/45	15/5 yr	—
Cochran [56]	5(4)	36–44	9 mo–8 yr	—
Skworc [57]	5(3)	51–61	4 mo–8 yr	Plaque-like PrP aggregates with droplet-like structures
Beck [58]	5(1)	45	>4 yr	—
Mead [59]	5(5)	34–63	6 mo–12 yr	Kuru-like plaques, PrP deposition
Oda [60]	6(6)	25–36	4–10 yr	PrP plaques
Capellari [61]	6(3)	31–38	4–10 yr	Elongated PrP patches
King [62]	6(2)	31/37	8/15 yr	Dense patches of PrP deposits
Gelpi [63]	6(1)	65	4 yr	PrP globules
Kovacs [64]	6(2)	33/35	3/3 yr	Diffuse PrP deposits
Collinge [65], King [62]	6(28)	22–53	1–18 yr	PrP patches/amyloid
Owen [66]	6(1)	middle age	—	—
Goldfarb [14]	7(6)	23–35	—	—
Mizushima [67]	7(1)	29	4–13 yr	PrP plaques
Dermaut [68]	7(3)	24–32	11–17 yr	Elongated PrP deposits
Lewis [69]	7(1)	29	16 yr	Patchy PrP deposits
Wang [70]	7(1)	44	4 yr	—
Goldfarb [14]	8(4)	35–55	3 mo–5 yr	Multicentric (GSS) plaques
Goldfarb [71]	8(4)	37–46	6 mo–13 yr	Kuru-like and multicentric amyloid plaques
Van Gool [72], Stam [73]	8(6)	21–54	1–7 yr	Globular PrP deposits
Laplanche [74]	8(10)	21–42	15 mo–7 yr	Kuru and multicentric plaques
Owen [75]	9(1)	53	2 yr	—
Duchen [76]	9(1)	55	2.5 yr	Amyloid PrP plaques
Krasemann [77]	9(1)	32	—	—

**Figure 5:**
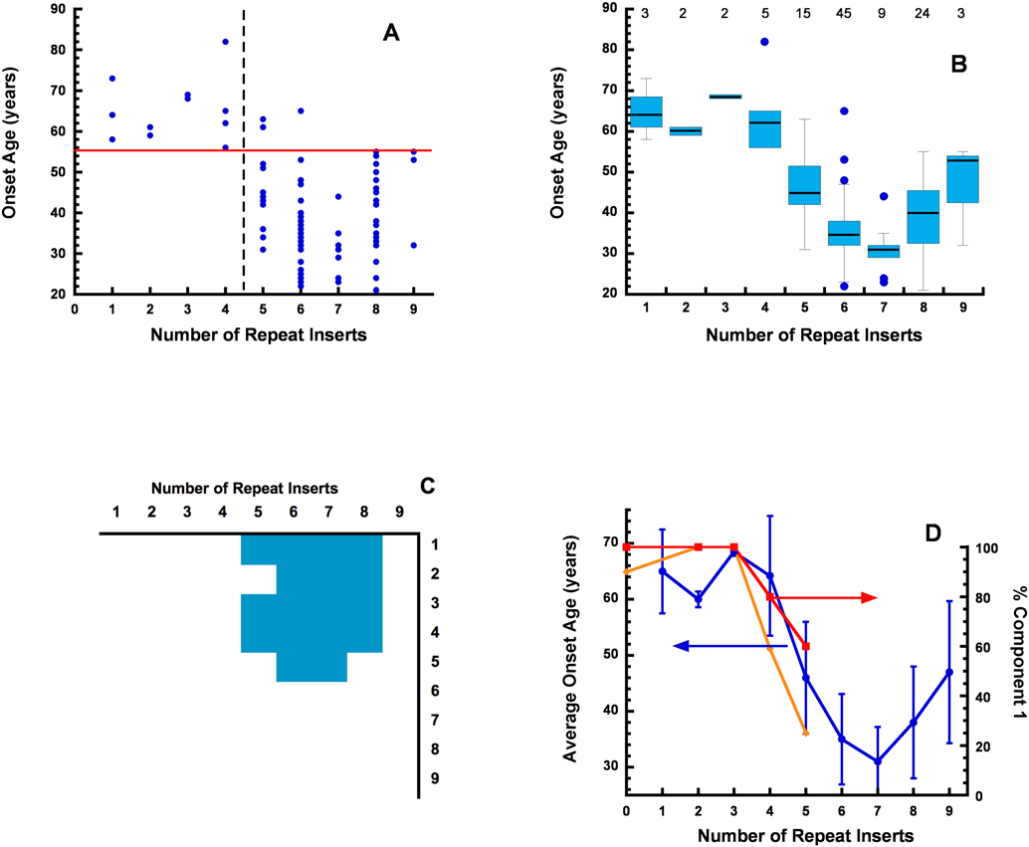
The relationships among OR length, onset age, and copper binding properties. (A) Onset age for individual cases as a function of extra octarepeat inserts. Note that wild-type corresponds to four repeats, so three inserts corresponds to seven total repeat segments, as in Figures 3 and 4. The horizontal line represents 55.5 years of age and the vertical line separates four and five inserts (separators determined by CART analysis). For all cases with four or fewer inserts (e.g., up to eight total repeats), onset age is more than 55.5 years; for five inserts and longer, 96% of the cases exhibit onset age younger than 55.5 years. (B) Parallel box plot of the data in (A), with the number of individuals in each group given at the top. Blue circles are outliers. (C) F test results for all pairwise comparisons based on disease onset age. Cells in blue correspond to significant pairwise differences, thus establishing groupings in onset age for 1–4 OR inserts (late onset) and for 5–8 inserts (early onset). (D) Average onset age, with standard deviation (blue circles, left axis), and component 1 coordination (orange diamonds and red squares, right axis, for 3.0 and 4.0 equivalents Cu^2+^, respectively) as a function of extra octarepeat inserts. At both copper concentrations, component 1 coordination drops suddenly at approximately the same OR length threshold as average onset age.

To examine these data more closely, we plotted the age of onset for each individual case against the number of OR insertions in Figure 5A. The red horizontal line is at 55.5 years (see below). All cases up to four OR inserts are above this line and 96% of the cases of five or more OR inserts are below the line. Although there is significant scatter in reported onset age for each specific OR length, the dramatic shift to early onset disease between four and five inserts is apparent. Figure 5B presents the same data as parallel boxplots, with sample sizes (number of cases) in each boxplot given at the top of the graph. An overall F test provided strong evidence of differences between the mean onset ages for different numbers of repeats (p-value 2.8e-14) [33]. The results for all pairwise comparisons are summarized in Figure 5C; cells in blue correspond to significant pairwise differences at a family-wise error rate of 5%. The results are consistent with the existence of two groups, one made of individuals with 1 to 4 OR inserts and another made of individuals with 5 to 8 inserts. The three patients with 9 repeats did not show a significant difference with any of the other groups; this is due to the small sample size in that group.

These results were further supported by an analysis via regression trees and cubic regression (see Protocol S1). Classification and regression trees (CART) [34] introduce binary cuts in the predictor variable (in this case, number of OR insertions) in a way that maximizes the distance (measured in terms of the outcome variable, in this case onset age) between the two groups, with cross-validation to ensure that spurious splits are not identified. When CART was applied to our data, a single split was found, dividing the data set into two groups: patients with 1 to 4 OR inserts (mean onset age of 64.4 years) and patients with 5 to 9 inserts (mean onset age of 37.9 years, which clearly differs from 64.4 years by an amount which is large in clinical/biological terms). Similarly, all non-constant terms in a cubic regression of onset age on number of repeats were highly significant (p<0.0001), and the overall F-test for comparing the cubic model to a constant-age-of-onset model had a p-value of 4.4e-15. These results confirm a nonconstant relationship between the number of repeats and the onset age in the population of individuals similar to those in our data set.

CART was also employed to find an optimal onset-age separator between the group of low (1–4) and high (5–9) number of OR inserts (horizontal red line in Figure 5A). For this purpose, we treated the group membership as a dependent binary variable and used onset age as the independent variable; the optimal separator between the groups corresponded to an age of 55.5 years.

Disease duration and OR number are also related in a manner that is significant both statistically and clinically/biologically. In our analysis, duration rose almost monotonically from a mean of 0.4 years for one OR insert to a mean of 10.9 years for seven inserts, and then fell to a mean of 2.3 years for nine inserts (ANOVA p-value 7.3e-08 with log(duration) as the outcome; comparison of all pairs of means with multiplicity adjustment supported both the rise and fall just mentioned (see Protocol S1); p-value of 2.7e-06 from a cubic regression of log(duration) on OR number, testing the overall cubic model against a null model of no relationship; 27 cases set aside for missing duration data; additional analyses provided in Protocol S1). For cases of up to seven OR inserts, our results are consistent with those of Croes et al., who identified a strictly monotonic increase in survival time for individuals with more OR inserts [32]. For these cases, we cannot determine whether this strong positive relationship is a direct consequence of the specific PrP sequence or, alternatively, is due to older individuals succumbing more quickly to disease. However, a recent study of sporadic Creutzfeldt-Jakob disease cases found that young individuals who developed symptoms below the age of 50 lived almost three times longer than those who developed disease after 50 [35]. Moreover, in the younger group, disease duration was not influence by specific PrP genotype. As noted, our analysis reveals an interesting reversal of the general trend for cases of eight or more OR inserts: despite early onset disease, these individuals exhibit short disease duration more consistent with those who developed symptoms later in life. From the perspective of Cu^2+^ uptake, 12 repeats, corresponding to eight inserts, would likely be the threshold for 3.0 equivalents of component 3 coordination (four His for each of the three Cu^2+^ ions). It is possible that PrP with more than eight OR inserts exerts a rapid rate of neurodegeneration, perhaps due to yet further alterations in copper binding, that cannot be overcome even in youthful individuals.

The most striking relationship with OR insert number we identified is the age of onset, which shows a sudden drop between four and five inserts from 64.4 years to 37.9 years. Figure 5D compares the average onset age and standard deviation, as a function of OR length, to Cu^2+^ binding properties. As developed above, the longest OR expansions favor component 3 coordination and resist component 1. Thus, component 1 coordination serves as a convenient measure of altered Cu^2+^ binding properties. Figure 5D shows the relative population of component 1 coordination for each OR construct, as derived from our copper titrations above, superimposed on the average age of onset. For wild-type and expansions involving up to seven ORs (three inserts beyond wild-type), component 1 coordination is dominant for both 3.0 and 4.0 equivalents Cu^2+^. However, at eight and nine ORs (four and five inserts, respectively), the population of component 1 coordination drops precipitously. For example, at 3.0 equivalents Cu^2+^, component 1 coordination is nearly 100% for three inserts and drops to approximately 25% for five inserts. Experimental challenges with solid phase synthesis and protein expression prohibited the study of yet longer OR sequences in either polypeptide constructs or full-length protein, respectively; however, the trend to favor component 3 for long inserts is clear and would not reverse for six OR inserts and beyond. Thus, Cu^2+^ coordination shows a transition between four and five inserts, coincident with the OR length that correlates with early onset prion disease.

## Discussion

The wild-type OR domain with four repeats responds to increasing copper concentrations by transitioning from component 3 to component 1 coordination. Our data show that this process is preserved in longer OR domains up to seven total PHGGGWGQ repeats. However, for eight repeat segments (four inserts beyond wild-type) and beyond, this transition is significantly inhibited. The biophysical basis for this likely arises from the number of repeats required for component 3 coordination. As demonstrated in our previous work, component 3 involves coordination of approximately four His side chains from adjacent octarepeat segments. If four repeats are required, then OR domains of up to seven total repeats may only take up a single Cu^2+^ in the component 3 binding mode, as observed. However, eight total repeats allows for two equivalents of component 3 coordination. Thus, higher copper levels are required to drive the transition to component 1 coordination. These arguments based on the stoichiometric ratio of OR segments to copper are straightforward. However, an unexpected finding is that expanded OR domains with more than eight repeats exhibit an approximate 10-fold increase in Cu^2+^ binding affinity. This affinity shift, in concert with His side chain counts favoring two equivalents of component 3, contributes to the decrease in component 1 coordination for OR domains with four or more inserts beyond wild-type.

**Figure 6:**
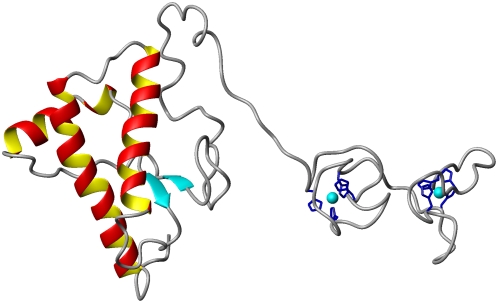
Three-dimensional model of PrP with eight total repeats (four inserts) coordinated to two equivalents of Cu^2+^ in the component 3 mode. (Note that N-terminal residues 23–59 are not shown.) Results presented here demonstrate that this structure is persistent and, in contrast to wild-type PrP, resists transitioning to component 1 coordination (Figure 1).

To gain insight into the three-dimensional characteristics of PrP with an expanded OR domain interacting with Cu^2+^, we performed structure calculations using distance restraints tethering four adjacent repeat His side chains to a single copper ion. We examined PrP with eight repeats and two copper equivalents. The C-terminal domain coordinates are from NMR studies. Other than Cu^2+^-imidazole distances, the OR domain was left unrestrained during energy minimization. The resulting structure is shown in Figure 6. (Non-octarepeat Cu^2+^ are omitted and approximately 40 residues on the N-terminal side of the first repeat are not shown.) His imidazoles arrange with an approximate tetrahedral geometry around each copper center. As expected, the expanded OR domain readily takes up two Cu^2+^ with a relaxed backbone conformation. Also, with eight total repeat segments, the OR domain comprises a significant fraction of the total protein. Although each copper center carries a divalent positive charge, the rest of the 64 amino acids within the expanded OR domain are uncharged and thus comprise a significant hydrophobic domain. PrP with OR inserts show a strong propensity to form aggregates and amyloid. The enhanced hydrophobicity of the N-terminal domain may facilitate interactions between PrP^C^ copies, thus promoting the amyloid assembly process.

To explore the effect of OR inserts on amyloid formation, Dong et al. developed a chimeric Sup35 yeast protein, in which PrP octarepeats replaced the endogenous repeat sequences [28]. Amyloid fibers assembled spontaneously from chimeras containing both 4 and 8 repeats but, in unseeded reactions, the lag time was substantially shorter for the chimera with the longer OR domain. Interestingly, when Cu^2+^ was added in proportion to the number of repeats in each chimera, the lag time decreased in the 4 OR construct but increased in the 8 OR construct. Leliveld et al. examined glutathione-S transferase (GST) fusion proteins onto which OR domains of varying length were grafted to the protein C-terminus [16]. Longer OR constructs exhibited enhanced multimerization and, at a threshold of 10 ORs, an ability to directly bind PrP^Sc^. Copper promoted multimerization in both short and long OR constructs, but the longer OR domains also exhibited irreversible aggregation in the absence of copper. In contrast, new studies of expanded mouse PrP suggest that OR inserts actually decrease amyloid production [17].

The OR length also correlates with the progression of prion disease. Expansions of up to four additional repeats gives the phenotype of familial Creutzfeldt-Jakob disease (fCJD), characterized by PrP^Sc^ deposits in the cerebral cortex and associated dementia [5]. For OR domains containing more than four inserts, the presentation is consistent with Gerstmann-Straussler-Scheinker disease (GSS), in which deposits are concentrated in the cerebellum and individuals suffer from ataxia. Amyloid is common in GSS but our review of disease associated with OR expansion, regardless of length, finds most cases reporting plaques and amyloid (Table 2).

Progressive elongation of the OR domain leads to alterations of PrP's molecular properties, with influence on the tendency to aggregate, but there must be an additional mechanism responsible for the sudden and profound shift in age of onset observed between four and six inserts. As derived from data in Table 2, the average age of onset for four, five and six inserts is 64, 47 and 34, respectively. Thus, addition of two repeats lowers the onset age by 27 years. Sequence analysis of the OR domain suggests that both component 3 and component 1 coordination are physiologically important [22]. Component 3 coordination requires four OR His residues, a count that is almost perfectly conserved for all mammalian species (several species have five repeats). Alternatively, component 1 coordination does not depend on the number of repeat modules but instead on the specific HGGGW segment within each repeat. Again, this sequence is completely conserved (except for the third Gly, which is Ser in mouse). Our data demonstrate a profoundly shifted equilibrium between component 3 and component 1 coordination for an OR domain of eight or more repeats (four or more inserts) that directly correlates with the observed lowering in onset age.

These findings point to altered copper binding in lowering the onset age for prion disease. We consider three possible causes. First, loss of component 1 coordination may lead to enhanced redox stress. As analyzed in our papers and elsewhere, component 1 coordination stabilizes copper in the Cu^2+^ oxidation state [21],[26]. Without complexation, copper cycles between Cu^+^ and Cu^2+^, contributing to the production of reactive oxygen species. Copper concentrations vary significantly in the synaptic space, a region of high PrP expression. At rest, synaptic copper concentrations are approximately 3.0 uM [36]. However, upon neuronal depolarization, copper is released from the presynaptic surface and the concentration elevates to approximately 250 uM [37]. The resting concentration is below the component 1 dissociation constant of 10 uM, indicating that component 3 coordination dominates. However, as the copper concentration rises, PrP reorganizes to take up Cu^2+^ in the component 1 mode. Thus, redox protection emerges at high copper levels. For PrP with more than four inserts, transition to component 1 coordination is inhibited, as indicated by the data in Figure 3, resulting in a loss of copper redox suppression. Also, with expanded OR domains showing a 10-fold increase in copper affinity, the off-rate allowing the component 3 to component 1 transition may be kinetically sluggish.

Another possibility is that an expanded OR domain interferes with the ability of PrP^C^ to interact with binding partners on the cell surface. Transgenic mice with alterations of the intervening sequence between the PrP OR domain and the folded C-terminus show significant neuronal degeneration [38],[39]. Current thinking suggests that PrP^C^ interacts in a bivalent fashion with a receptor that plays a role involving cellular signaling or regulation. A possible binding partner candidate is the low-density liproprotein receptor related-protein 1, LRP1 [40]. Interaction with LRP1 is required for copper mediated PrP^C^ endocytosis, and either elimination of the OR domain or elongation to 14 total repeats completely halts PrP^C^ cycling. These findings point to a copper-dependent conformational change in the OR domain consistent with the component 3 to component 1 transition.

The third possibility considers the role of copper in the conversion of PrP^C^ to PrP^Sc^. Using protocols for producing synthetic prions from recombinant mouse PrP, Bocharova et al. showed that Cu^2+^ increases the lag time for conversion to amyloid [41], an effect similar to that observed in the Sup35 chimera with eight repeats [28]. At a 1∶1 copper to protein ratio, influence on the lag time was minimal. However, a 10-fold higher copper concentration resulted in a lag time increase of over 100%. Copper also inhibited polymerization of PrP(89–230), lacking the OR domain, but the effect was less pronounced. The high ratio of copper required to inhibit amyloid suggests that component 1 coordination is effective at protecting against polymerization. As noted above, the 8 OR Sup35 construct exhibited increasing lag times at near saturating copper levels [28]. In this scenario, PrP^C^ with eight or more repeats resists component 1 coordination and is therefore more susceptible than wild-type to misfolding as amyloid. This is certainly consistent with the widespread amyloid observed in GSS resulting from repeat inserts.

In summary, our findings demonstrate a very strong relationship between changes in copper binding properties and early onset prion disease. The role of the octarepeats in prion disease is enigmatic. Although the OR domain is not part of the protease resistant scrapie particle, it nevertheless modulates disease progression. The studies here point to contributing factors in prion neurodegeneration, and suggest either loss of PrP^C^ copper protein function or loss of copper-mediated protection against conversion to PrP^Sc^.

## Materials and Methods

### Peptide Synthesis and Purification

All peptides were synthesized using fluorenylmethoxycarbonyl (Fmoc) methods, as previously described [27],[42]. N-terminal acetylation and C-terminal amidation were included to avoid non-native backbone charges. Peptides were purified by reverse-phase HPLC and characterized by mass spectrometry.

### Protein Expression and Purification

Syrian Hamster PrP (rPrP(23–231)) was expressed in the pET101 vector (Invitrogen) in *E. coli* BL21 Star (DE3) cells (Invitrogen), as previous described [29]. The protein was solubilized from inclusion bodies with 8 M urea (pH 8) and flowed over a nickel charged immobilized metal affinity chromatography (IMAC) column. The protein was eluted from the column with pH 4.5 urea. Protein folding was achieved by raising the pH to 8.5 and desalting with a column of Sephadex G-25 (HiPrep, Amersham). The folded protein was then repurfied by HPLC, characterized by mass spectrometry and lyophilized. Correct protein fold was confirmed by circular dichroism. As previously described, mutant mouse PrP with a total of nine repeats (rPrP+5OR) was generated by multiple rounds of PCR based mutagenesis followed by expression using the pET23 vector [43]. Recombinant PrP+5OR was purified using a copper charged IMAC column [44]. Additional purification and characterization followed treatments similar to those applied to rPrP(23–231). Although both hamster and mouse sequences were used, we note that amino acid sequences in the copper binding segments and measured EPR, affinity and coordination modes are equivalent between the two wild-type (four repeat) proteins.

### Electron Paramagnetic Resonance (EPR) Spectroscopy

All samples were prepared with buffer containing 37.5 mM MOPS and 18.75% glycerol (v/v), as a cryoprotectant, with pH adjusted to 7.4 [42]. X-band spectra (frequency of 9.43 GHz, microwave power of 1 mW and modulation amplitude of 5.0 G) were acquired at 125 K using a Bruker EMX spectrometer with an SHQ cavity (Bruker) and a variable temperature controller. Sample spectra were fit to basis spectra using non-negative least-squares (NNLS) routines in the Matlab program suite, as previously described [19].

### Structure Calculations

Calculations for two equivalents of copper loaded into eight octarepeats as component 3 were calculated using the CYANA torsional dynamics program [45]. The copper ions were placed 2.01Å from the Nδ atom of four histidine residues. Two copper ions were modeled into the sequence (PHGGGWGQ)_8_GGGTH. The first four histidine residues were linked to the first copper ion and the second copper ion was bound to the next four histidine residues. Calculations were performed that maintained fixed peptide bond distances and angles. Upper and lower limit distance restraints between the copper atom and the histidine residues were used to calculate the model structure. 100 structures were calculated and the lowest energy conformer was retained. The model was then linked to PDB coordinates from PrP(97–231) to create the full-length model structure [46].

### Statistical Analysis

For a formal analysis of the relationship between the number of octarepeat inserts and the onset age, we used a standard one-way ANOVA model in which the independent variable corresponded to the number of repeats (treated as a categorical variable) and the dependent variable corresponded to the onset age. To account for multiplicities, multiple comparisons were performed using Tukey's adjustment [33]. Other data-analysis tools employed include classification and regression trees (CART) [34], which were used to confirm the results of the ANOVA model and to determine the separating age between early and late-onset disease, and polynomial regression, which served to strengthen the results from the ANOVA model by treating the number of octarepeats quantitatively. All calculations were performed in the statistical computing environment R (http://www.r-project.org). Complementary analyses, arriving at similar conclusions using nonparametric regression, the bootstrap, and Bayesian change-point modeling, are available in the online Protocol S1.

## Supporting Information

Protocol S1 Statistical analysis using nonlinear regression and Bayesian change point models. These findings provide additional support for the relationship between OR length and onset age, and also reveal evidence of shortened disease duration in cases of eight or more inserts.
(0.20 MB PDF)
