# Supplementary material for: Early Onset Prion Disease from Octarepeat Expansion Correlates with Copper Binding Properties
Source: PLoS Pathog. 2009 Apr 17;5(4):e1000390. doi: 10.1371/journal.ppat.1000390 (PMC2663819; doi:10.1371/journal.ppat.1000390)
Supplement: Protocol S1 — Statistical analysis using nonlinear regression and Bayesian change point models. These findings provide additional support for the relationship between OR length and onset age, and also reveal evidence of shortened disease duration in cases of eight or more inserts. (0.20 MB PDF) [file ppat.1000390.s001.pdf]

Supplement to "Early onset prion disease from octarepeat expansion correlates with copper binding properties," by Stevens DJ, Walter ED, Rodríguez A, Draper D, Davies P, Brown DR, Millhauser GL

1 Supplemental analyses relating age at onset of disease to number of octarepeats (OR)

In addition to the analysis of variance and regression results presented in the main paper, we performed two additional analyses examining how age at disease onset is related to the number of octarepeats (OR). In the first of these analyses, we used a frequentist nonlinear regression method to characterize the mean age as a function of OR number; the second analysis fitted a Bayesian change-point model to the same relationship.

Table 1 (Supplement) gives a descriptive summary of onset age at each observed level of OR number, and Figure 1 (Supplement) presents a scatterplot of age versus OR number, with a cubic regression fit (corresponding to one of the analyses in the main paper), a nonparametric smooth — based on the `lowess` function (Cleveland, 1979) in the statistical analysis environment `R` (R Development Core Team, 2003) — and a constant model (at the mean age of 40.2 years) superimposed. This graph is highly suggestive of a strong non-constant relationship between the two variables, in which — as the number of octarepeats increases — the mean onset age at first declines and then rises again. This can be confirmed by bootstrapping (e.g., Efron and Tibshirani, 1993) the `lowess` function, as in Figure 2 (Supplement); the vertical envelope of dotted lines in the figure for any OR number provides an approximate 99% nonparametric interval estimate for the median age at onset for that OR number. It is clear that the underlying relationship in the population of individuals similar to those in our study is non-constant.

The problem of testing for a difference in the onset age among individuals with different numbers of octarepeats can also be tackled using a model that assumes that the onset age is a piecewise-constant function of the OR number, possibly with one single change-point after an undetermined number of repeats. As an alternative analysis we also fit this model, commonly known as a single change-point model, using Bayesian methods (e.g., Robert, 2007), which require the elicitation of prior distributions that, combined with the information in the data, produce posterior probability distributions upon which inference is based.

Table 1: *A descriptive summary of onset age at each observed level of OR number.*

	Onset Age (Years)		
OR Number	Mean	Standard Deviation	Frequency
1	65.0	7.5	3
2	60.0	1.4	2
3	68.5	0.7	2
4	64.2	10.7	5
5	46.9	10.0	15
6	33.6	9.8	45
7	30.9	6.2	9
8	38.8	10.0	24
9	46.7	12.7	3
Overall	40.2	13.6	108

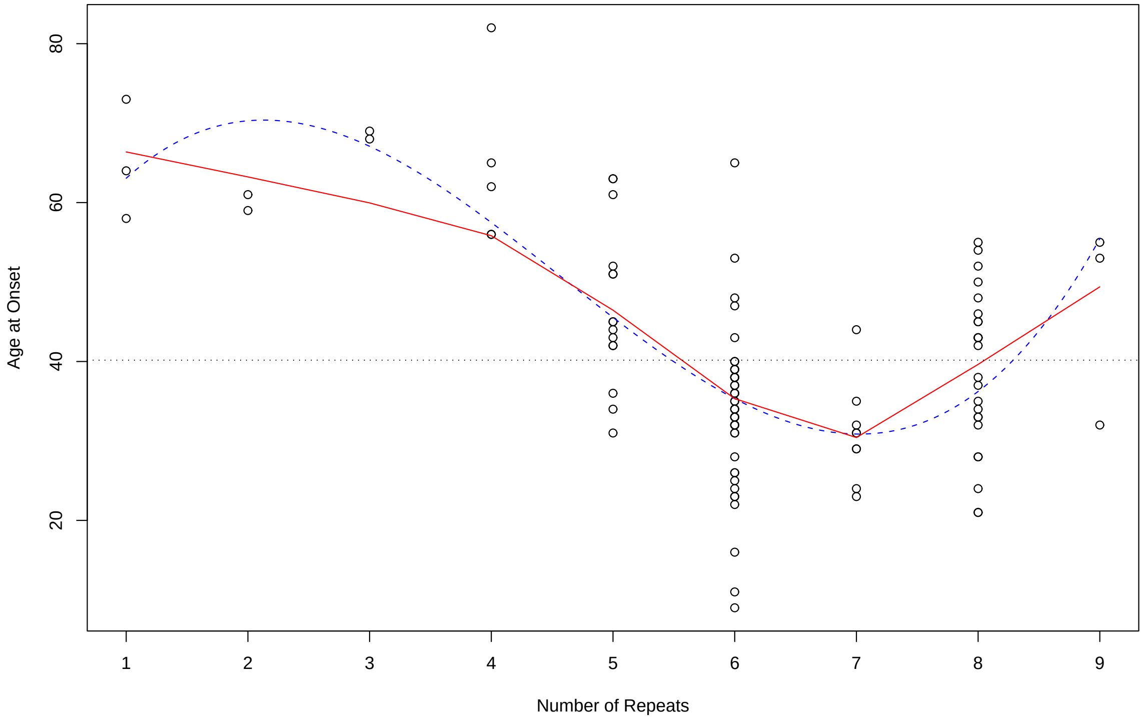

Figure 1: *Age at onset as a function of number of repeats, with a cubic regression fit (blue dotted curve) and a nonparametric smooth (red solid curve) superimposed; the constant model (at the mean age) is given (black short dotted line) for comparison.*

In brief, our model assumed that the patients are divided in up to two groups (above and below an unknown number of repeats) and that the onset ages within each group follow a Normal distribution with unknown parameters. With 9 observed OR number values in our data set, testing for differences and identifying the unknown change-point effectively reduced

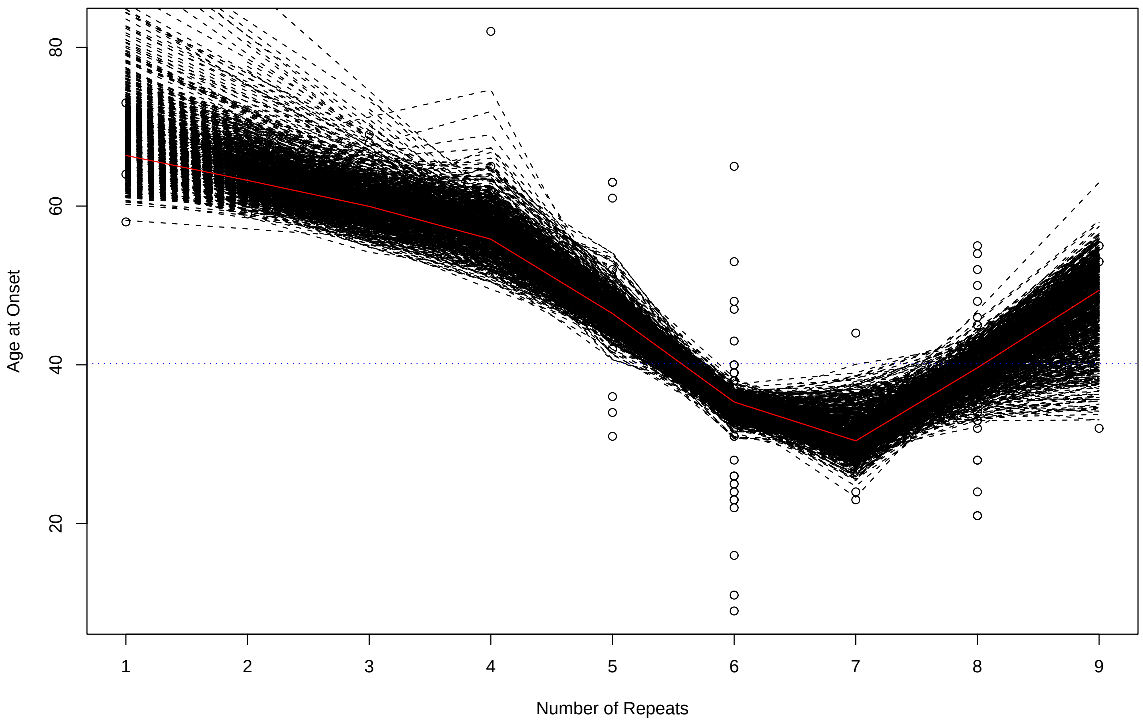

Figure 2: *Scatterplot as in Figure 1 (Supplement), with 1,000 bootstrap* `lowess` *curves superimposed.*

to comparing 9 models: the model that assumes no change point in the data (to which we assigned a prior probability of $\frac{1}{2}$, and the 8 models corresponding to a change-point after each OR number (each of which was given a prior probability of $\frac{1}{16}$, which means that, a priori, we think that a change point has the same probability of occurring after any number of repeats). For the other unknown parameters in the model (the mean and variance of each group) we employ conjugate Normal-Inverse-Gamma priors, with parameters chosen to reflect historical information from previous studies. The priors we used in this analysis implied that the average onset age has a mean of 50 years, and that we expect it to be between 0 and 100 years with roughly 95% probability. We further assumed that the standard deviation of the onset age within each group is around 12 years and has a 95% probability of being below 25 years.

With these conjugate priors, closed-form expressions for the posterior probabilities of the 9 models involved can readily be obtained, allowing us to avoid Monte Carlo integration (the calculations are straightforward and are therefore omitted). The posterior probability (given the data) for the model with no change point was $1.3 \cdot 10^{-12}$, which provides decisive evidence against a model in which onset age does not depend on OR number. (The fact that this probability is so small also makes it clear that even dramatic changes, for sensitivity analysis purposes, in the prior probabilities for the no-change-point and one-change-point models would have no effect on this conclusion.) In terms of the location of the change-point, there was decisive evidence of a change in the average onset age after either 4 or 5 repeats, but the exact location of the change-point was not completely clear: the posterior probability

Table 2: *A descriptive summary of disease duration at each observed level of OR number.*

	Disease Duration (Years)		
OR Number	Mean	Standard Deviation	Frequency
1	0.4	0.1	3
2	7.0	0.0	1
3	1.7	1.9	2
4	1.2	1.8	4
5	5.3	4.9	11
6	7.4	4.1	32
7	10.9	4.3	8
8	3.4	3.1	18
9	2.3	0.4	2
Overall	5.7	4.6	81

for a change between 4 and 5 repeats was 0.72, while the probability of a change between 5 and 6 repeats was 0.27 (all other locations for the change point had posterior probabilities of less than 10^{-6}). Sensitivity analysis showed that, overall, these results were fairly robust to moderate changes in the prior distribution; for example, a prior mean for the average onset age of around 40 years roughly evened out the posterior probabilities of the change-points at 4/5 or 5/6. These results are broadly consistent with those we described in the main text of the paper and with the results presented earlier in this section.

2 Supplemental analyses relating duration of disease to OR number

We also performed additional analyses examining the relationship between disease duration and octarepeat number. Table 2 (Supplement) presents a descriptive summary of disease duration at each observed level of OR number; disease duration was heavily positively skewed, suggesting a logarithmic transformation. The top panel in Figure 3 (Supplement) gives a scatterplot of the logarithm of disease duration as a function of number of repeats, with a cubic regression fit (corresponding to one of the analyses in the main paper) and a nonparametric smooth (red solid curve) superimposed, and with a constant model at the mean log duration (1.3) for comparison. Once again it is evident that the relationship is strongly non-constant; once again, to confirm this the `lowess` nonparametric smooth can be bootstrapped, as in the bottom panel in Figure 3 (Supplement). Here there is more vertical uncertainty than in Figure 2 (Supplement), but significant departures from a constant model are again evident.

As noted in the main text, we used an ANOVA of log(duration) on OR number, followed by multiple-comparison-adjusted pairwise comparisons of means, to find statistically significant

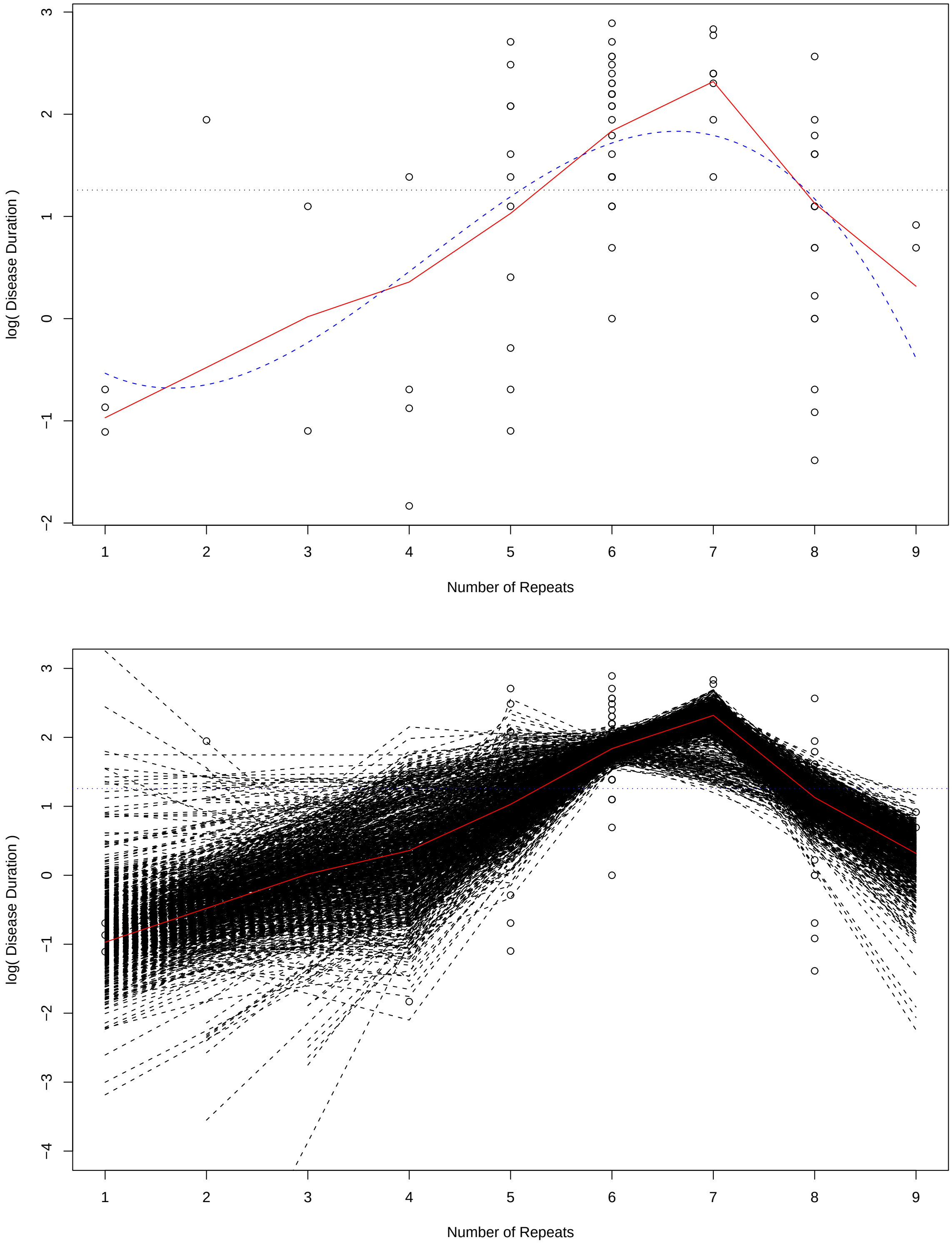

Figure 3: *Top panel: Logarithm of disease duration as a function of number of repeats, with a cubic regression fit (blue long dotted curve) and a nonparametric smooth (red solid curve) superimposed; the constant model (at the mean log duration) is given (black short dotted line) for comparison. Bottom panel: Scatterplot as in top panel, with 1,000 bootstrap* `lowess` *curves superimposed.*

Table 3: *Summary of all significant pairwise comparisons with log(duration) as the outcome and OR number as a categorical predictor.*

Moving from	1	to	5	repeats, disease duration goes	up.
	1		6		up.
	1		7		up.
	3		7		up.
	4		6		up.
	4		7		up.
	6		8		down.
	7		8		down.

differences in the relationship between disease duration and number of octarepeats. We conclude with a summary, in Table 3 (Supplement), of the pairwise differences found significant at the 0.05 level by the Tukey-Kramer HSD method.

References

Efron B, Tibshirani RJ (1993), *An Introduction to the Bootstrap.* New York: Chapman & Hall.

Cleveland WS (1979). Robust locally weighted regression and smoothing scatterplots. *Journal of the American Statistical Association*, **74**, 829–836.

R Development Core Team (2003). *R Reference Manual: Base Package*, Volume 1. Bristol UK: Network Theory Ltd.

Robert C (2007). *The Bayesian Choice: From Decision-Theoretic Foundations to Computational Implementation.* New York: Springer Verlag.
